# Chemotherapy-Induced Neutropenia in Pediatric Leukemia: Persian Medicine Perspective

**DOI:** 10.31661/gmj.v8i0.1600

**Published:** 2019-10-31

**Authors:** Babak Daneshfard, Mahdi Shahriari, Majid Nimrouzi

**Affiliations:** ^1^Student Research Committee, Shiraz University of Medical Sciences, Shiraz, Iran; ^2^Hematology Research Center, Namazi Hospital, Shiraz University of Medical Sciences, Shiraz, Iran; ^3^Department of Persian Medicine, School of Medicine, Shiraz University of Medical Sciences, Shiraz, Iran

**Keywords:** Chamomile, Neutropenia, Chemotherapy, Leukemia, Persian Medicine

## Dear Editor,

With lots of interest, we read the paper by Aminian and Yousefi [1]. They had discussed the concept of chemotherapy-induced nausea and vomiting (CINV) in Persian medicine (PM) and compared it with the modern medicine viewpoints. Other than CINV, chemotherapy-induced neutropenia (CIN) is a common complication of treatment in cancer patients. Cancer is a leading cause of death worldwide. Leukemia is the most common type of malignancy in children, among which acute lymphoblastic leukemia (ALL) is the most prevalent one [2]. Although chemotherapy has dramatically improved the long-term survival of the patients [3], it itself could be the cause of various complications including neutropenia. CIN is hematologic toxicity, which in turn increases the probability of infection, duration of hospitalization, and the need for antibiotics and granulocyte colony stimulating factor (GCSF) consumption. Nowadays, complementary and alternative medicine (CAM) is commonly used by children with cancer [4]. In this regard, herbal medicine as a popular branch of CAM with a very long history of usage [5], has shown a great potential for anti-cancer treatment. Moreover, some of the well-known anti-cancer drugs such as Vincristine and Vinblastine are plant-derived [6]. These FDA approved drugs are commonly used in chemotherapy regimen of ALL and other malignancies. PM is a *Hikmat* (philosophy)-based holistic medical system stands on the theory of temperament and humors [7]. It has its own specific approach towards diagnosis and treatment of the diseases in which, phytotherapy has an important place. “*A’ya*” is a term in PM for a diseased state presents with clinical manifestations such as fatigue, bone/joint pain, and fever [8]. These presentations resemble that of the initial stage of leukemia. This condition is attributed to a kind of plethora of humors/materials in the body. In this situation, *Hararat-e-gharizi* (innate heat) is unable to dissolve or transfer the excess material due to its weakness [9]. In this regard, chamomile has been introduced in PM as a very useful drug for “*A’ya*” so that no other drug has such an effect. This benefit of chamomile is attributed to its boosting effect on innate heat [10]. *Matricaria chamomilla* L. (Asteraceae), known as German chamomile, possesses several anti-cancer and chemo-protective ingredients including apigenin [11], chamazulene [12], and bisabolol oxide A [13]. Therefore, it is not surprising that chamomile has been known as a safe anti-cancer and immunomodulatory herb. Results of our recent clinical investigation in our referral center for pediatric oncology revealed that chamomile could reinforce the immune system in children with ALL undergoing chemotherapy. It significantly increased the absolute neutrophil count and consequently, decreased the rate of neutropenia. Other than herbal therapy with chamomile that could be prescribed as a complementary treatment in leukemic patients, PM has useful suggestions regarding their diet and lifestyle modification which can improve the quality of life and increase the response rate to treatment. However, future clinical studies are needed to prove these effects.

## Acknowledgment

This paper has been derived from the Ph.D. thesis written by Dr. Babak Daneshfard sponsored by Shiraz University of Medical Sciences (grant number: 95-01-01-12564).

## Conflict of Interest

The authors have no conflict of interest.
